# Mirror-assisted light-sheet microscopy: a simple upgrade to enable bi-directional sample excitation

**DOI:** 10.1117/1.NPh.11.3.035006

**Published:** 2024-08-07

**Authors:** Asaph Zylbertal, Isaac H. Bianco

**Affiliations:** University College London, Department of Neuroscience, Physiology & Pharmacology, London, United Kingdom

**Keywords:** calcium imaging, light-sheet microscopy, selective plane illumination microscopy, volumetric imaging, zebrafish

## Abstract

**Significance:**

Light-sheet microscopy is a powerful imaging technique that achieves optical sectioning via selective illumination of individual sample planes. However, when the sample contains opaque or scattering tissues, the incident light sheet may not be able to uniformly excite the entire sample. For example, in the context of larval zebrafish whole-brain imaging, occlusion by the eyes prevents access to a significant portion of the brain during common implementations using unidirectional illumination.

**Aim:**

We introduce mirror-assisted light-sheet microscopy (mLSM), a simple inexpensive method that can be implemented on existing digitally scanned light-sheet setups by adding a single optical element—a mirrored micro-prism. The prism is placed near the sample to generate a second excitation path for accessing previously obstructed regions.

**Approach:**

Scanning the laser beam onto the mirror generates a second, reflected illumination path that circumvents the occlusion. Online tuning of the scanning and laser power waveforms enables near uniform sample excitation with dual illumination.

**Results:**

mLSM produces cellular-resolution images of zebrafish brain regions inaccessible to unidirectional illumination. The imaging quality in regions illuminated by the direct or reflected sheet is adjustable by translating the excitation objective. The prism does not interfere with visually guided behavior.

**Conclusions:**

mLSM presents an easy-to-implement, cost-effective way to upgrade an existing light-sheet system to obtain more imaging data from a biological sample.

## 1 Introduction

Light-sheet fluorescence microscopy is a powerful imaging tool with a large variety of bioscience applications.^1^ It is based on exciting the sample with a thin light sheet while imaging the emitted fluorescence via a perpendicular collection path. By restricting illumination to a single plane, light-sheet microscopes achieve optical sectioning while minimizing bleaching and photodamage. In a typical digitally scanned light-sheet setup, the sample is illuminated by a Gaussian beam that is rapidly scanned along the “y-axis,” perpendicular to the beam direction, using a galvanometric mirror to excite a single sample plane. For volumetric imaging, multiple planes are exposed sequentially by displacing the light sheet along the “z-axis” using a second scan mirror.

A particularly successful application of light-sheet microscopy has been *in vivo* cellular-resolution functional imaging in the small, transparent brains of larval zebrafish expressing fluorescent calcium indicators,^2^ which has provided insights into the function of distributed neuronal circuits controlling vertebrate behavior.^3^^–^^6^ In such experiments, the brain is illuminated from the side (laterally), and a light sheet is formed by scanning the beam in a direction (“y”) that corresponds to the rostrocaudal anatomical axis. However, in this arrangement, one of the eyes occludes the excitation beam, so a significant proportion of the total brain volume (∼20% to 25%^2^) cannot be imaged.

Existing solutions to this problem include changing the geometry of the excitation path with respect to the animal and/or adding a second path. Specifically, in dual-view selective plane illumination microscopy,^7^ both the excitation and detection axes are coherently rotated by 45 deg, and in swept confocally-aligned planar excitation microscopy,^8^ a single objective is positioned above the animal functions for fluorescence detection and sample excitation via an oblique plane. In other microscopes, a second excitation path has been constructed to illuminate the animal from an orthogonal “frontal” aspect, in addition to lateral illumination.^9^ Although these variants of light-sheet microscopy improve optical access, those utilizing oblique illumination require a completely new instrument design and cannot be easily implemented on existing setups. The addition of a second excitation path may be undesirable as it requires additional space for a second excitation objective (ExO), which hinders the presentation of visual stimuli in the animal’s frontal visual field. Such stimuli are commonly used, for example, in the context of virtual hunting assays.^6^^,^^10^

**Fig. 1:**
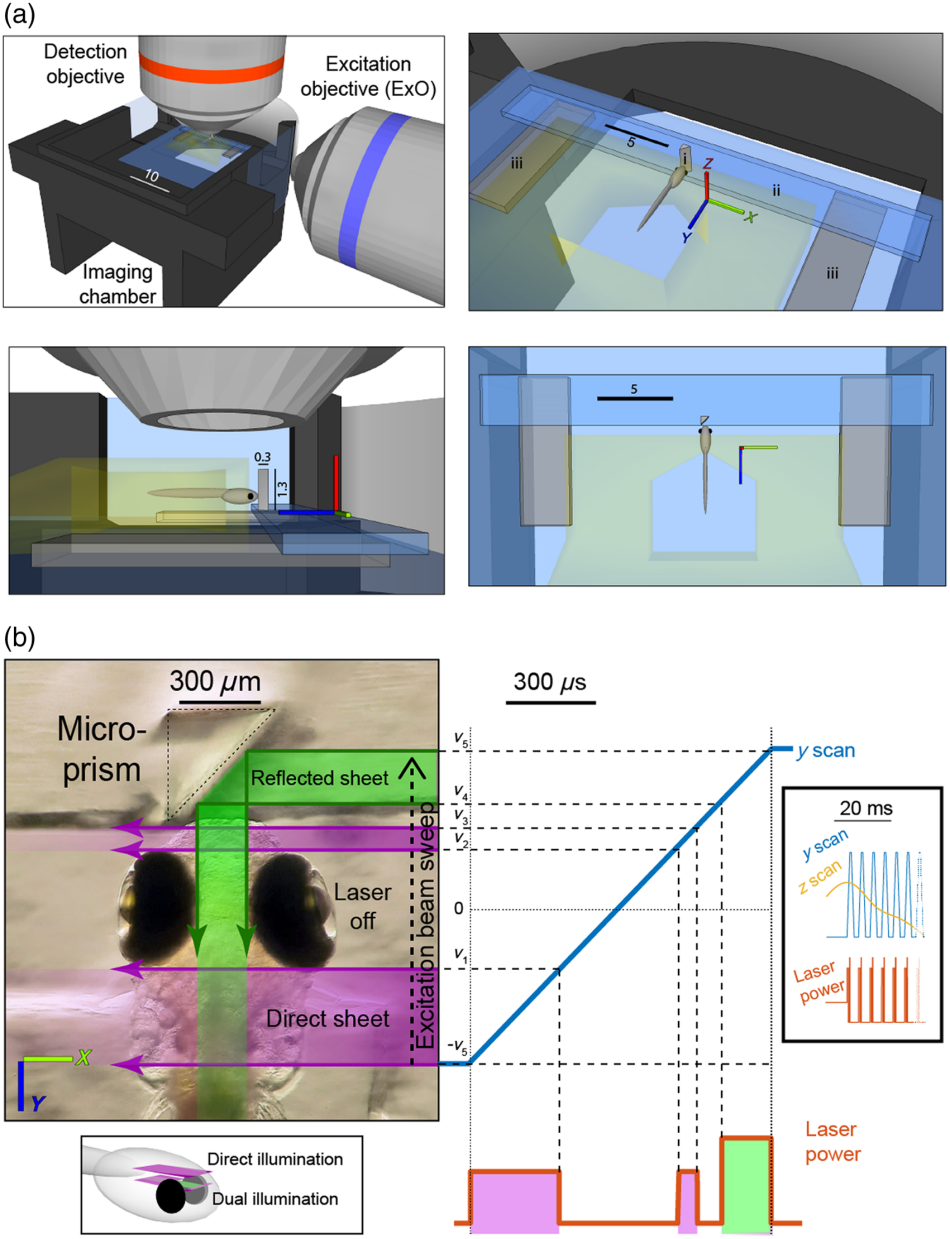
Overview of mLSM. (a) Schematic of the imaging chamber and the orthogonal excitation and detection objectives (top-left); isometric (top-right), side (bottom-left), and top (bottom-right) views of the fish and prism (i), glass strip (ii), and plastic bases (iii). Glass is depicted in blue and agarose in yellow; distances are indicated in millimeters. (b) Left: Zebrafish larva and micro-prism, overlaid with excitation laser paths for exposure of a dual illumination plane. Right: Analog voltage waveforms for y-mirror (blue) and laser power (orange) for imaging of a dual illumination plane. Magenta and green shading indicate direct (lateral) and reflected (frontal) sheets, respectively. $v_{1-5}$—y-mirror voltages corresponding to the posterior and anterior edges of the eyes ($v_{1/2}$), the rostral edge of the brain ($v_{3}$), and the medial surfaces of the eyes ($v_{4/5}$). Bottom inset: diagrammatic location of representative direct and dual illuminated planes. Right inset: Analog voltage waveforms for a volume scan, including multiple scan planes and the z-mirror waveform (yellow).

Here, we introduce mirror-assisted light-sheet microscopy (mLSM) as a simple and cost-effective solution that is easily implemented on existing light-sheet setups that employ unidirectional illumination. It is based on producing a second illumination path using a mirrored micro-prism, with corresponding adjustments to beam scanning and laser power modulation (Fig. 1). We demonstrate that this enables imaging of regions normally obscured during unidirectional illumination. In addition, we show that the image quality may be adjusted by displacing the ExO and that comparable, near-cellular resolution data can be obtained from the tissue that is illuminated directly or via the reflected sheet. Finally, we show that the micro-prism does not interfere with larval zebrafish responding to stimuli in their frontal visual field.

## 2 Materials and Methods

### 2.1 Animals

Zebrafish (*Danio rerio*) larvae were reared on a 14/10 h light/dark cycle at 28.5°C. For all experiments, we used zebrafish larvae homozygous for the ${mitfa}^{w2}$ skin-pigmentation mutation^11^ and ${Tg(elavl3:H2B\text{-}GCaMP6s)}^{jf5Tg}$ transgene (ZFIN ID: ZDB-ALT-141023–2).^9^ All larvae were fed *Paramecia* from 4 dpf onward. Animal handling and experimental procedures were approved by the UCL Animal Welfare Ethical Review Body and the UK Home Office under the Animal (Scientific Procedures) Act 1986.

### 2.2 Light-Sheet Microscopy Setup

We implemented mLSM on a custom-built digitally scanned light-sheet microscope that was in routine use in the lab.^6^ The excitation path included a 488 nm laser source with an analog input for power modulation (OBIS 488-50 LX, Coherent, Santa Clara, California, United States), a pair of galvanometer scan mirrors (6210H, Cambridge Technology, Bedford, Massachusetts, United States), and an objective (Plan 4X, 4x/0.1 NA, Olympus, Tokyo, Japan), with its back aperture limited to 5.5 mm using an adjustable iris (for an effective NA of ∼0.06). One mirror was used to scan the beam along the y-axis to produce a light sheet during a single camera exposure, and the second mirror displaced the light sheet along the z-axis across frames. Zebrafish larvae were mounted such that the y-axis corresponded to the anatomical rostrocaudal axis and the z-axis to the dorsoventral axis.

The orthogonal detection path, parallel to the z-axis, comprised a water-immersion detection objective (XLUMPLFLN, 20x/1.0 NA, Olympus), a telecentric tube lens (TTL200MP, Thorlabs, Newton, New Jersey, United States, f=200  mm), two relay lenses (a 2× apochromatic objective, TL2X-SAP, Thorlabs, f=100  mm, and a 50 mm Ø achromatic doublet, G322303000, Excelitas Technologies, Waltham, Massachusetts, United States, f=120  mm) in a 4f configuration, and an sCMOS camera (Kinetix, Teledyne Photometrics, Tucson, Arizona, United States). For remote focusing,^12^ an electrically tunable lens (ETL, EL-16-40-TC-VIS-20D, Optotune, Dietikon, Switzerland) was installed between the relay lenses, conjugate to the back focal plane of the detection objective.

### 2.3 Prism Assembly Preparation

The core component enabling mLSM is a custom fabricated 0.3×0.3×1.3  mm right-angle prism (45 deg by 45 deg by 90 deg), with its hypotenuse surface coated with aluminum for external reflection (Fig. 1 and Fig. S1(A, i) in the Supplementary Material, Precision Optics Corporation, Gardner, Massachusetts, United States). To prepare it for use in imaging experiments, we first glued the prism upright on a 3×18  mm strip of glass (cut from an 18×18  mm microscope coverslip), using UV-cured optical adhesive [#61, Noroland, Jamesburg, New Jersey, United States, Fig. 1 and Fig. S1(A, ii) in the Supplementary Material]. The glass strip was glued to two 2×5×0.75  mm clear polyethylene terephthalate glycol (abbreviated PETG or PET-G) plastic sheets that function as bases for the prism assembly [Fig. 1 and Fig. S1(A, iii) in the Supplementary Material]. Each prism assembly was then used repeatedly in multiple imaging sessions.

### 2.4 Animal Mounting and Prism Positioning

Larval zebrafish were mounted at 5 dpf in 3% low melting point agarose (Sigma-Aldrich, St. Louis, Missouri, United States) on top of a 22×22  mm coverslip. They were allowed to recover overnight before imaging at 6 dpf. Prior to the imaging session, the prism assembly was attached to the mounting coverslip using a small amount of silicone grease that was applied to its plastic bases. The coverslip with the prism assembly was then moved to a custom 3D-printed chamber (SLS Nylon 12, 3DPRINTUK, London, United Kingdom), and the prism assembly position was fine-tuned to make sure that the prism is oriented correctly and placed as close as possible to the fish but without touching it [Figs. 1(a) and 1(b)]. The long dimension of the prism (1.3 mm, its “height” along the z-axis) enabled flexibility in the mounting depth of the fish. The orientation of the prism has fairly wide tolerance margins due to the short distance between the prism and the sample. In our experience, manual adjustment under a dissecting microscope using the fish body axis as a reference was sufficient to accurately position the prism assembly.

To fine-tune the geometry of the reflected light sheet prior to imaging, a DC bias voltage was sent to the “y” scan mirror to displace the beam onto the prism, enabling the inspection of its reflected portion (using diluted fluorescein placed in the bath). Small errors in the angle of the prism with respect to the base coverslip would often cause the reflected beam to slant with respect to the imaging plane. We diagnosed beam slanting by translating the beam waist (by moving the ExO along its optical axis) and re-focusing the detection objective and corrected it by pitching the imaging chamber about the x-axis using a rotation stage (alternatively, this may be achieved using coverslips stacked beneath the chamber, see Fig. S1(B) in the Supplementary Material).

### 2.5 Image Acquisition

All microscope control was implemented using custom LabVIEW (National Instruments, Austin, Texas, United States) routines.

To assess the image quality and make point-spread function measurements, we acquired volumetric image stacks of brains or fluorescent beads using stepwise changes in the z-mirror position and corresponding ETL optical power. Brain anatomical stacks were comprised of 185 planes spaced 0.93  μm apart, each obtained by averaging 20 images (130  μW laser power at sample, 1 ms exposure). Image stacks of fluorescent beads were comprised of 200 planes spaced 0.2  μm apart, each obtained by averaging 25 images (520  μW laser power at sample, 35 ms exposure).

For functional calcium imaging, we acquired image volumes by dynamically changing the ETL power and z-mirror position [see waveform in Fig. 1(b)] at 5 volumes per second. Each volume was comprised of 37 imaging planes spaced 3.6  μm apart. Each plane was exposed to 130  μW laser power for 1 ms.

### 2.6 Scan Waveform Adjustment

For dual illumination via direct and reflected light sheets, we adapted the voltage waveforms sent to the scan mirrors and the analog laser power control signal [Fig. 1(b)]. To accommodate variation in the precise geometry of the fish with respect to the prism across sessions, the software enabled fine-tuning of the waveform properties, with online feedback. First, while viewing an imaging plane dorsal to the eyes, we tuned the y scan amplitude [Fig. 1(b), $+/-v_{5}$], ensuring that the reflected sheet reached the medial surface of the eye proximal to the ExO. In our system, $v_{5}$ was typically ∼1.5  V, well within the available scan range. Next, we tuned the voltages corresponding to the rostral edge of the brain ($v_{3}$), the side of the reflected sheet distal to the ExO ($v_{4}$), and the approximate posterior and anterior edges of the eyes ($v_{1/2}$). Last, while viewing a plane close to the dorsal edge of the eyes, we tuned the z scan voltage corresponding to the dorsal edge of the eyes to divide the imaging planes into “direct illumination only’ planes and “dual illumination” planes.

For direct illumination planes, the laser was on during the entire sweep across the brain ($-v_{5}$ to $v_{3}$). For dual illumination planes, the laser power was modulated to generate the reflected sheet (laser on between $v_{4}$ and $v_{5}$) and blanked while scanning over the eye ($v_{1}$ to $v_{2}$). The laser power was also increased between $v_{4}$ and $v_{5}$ to compensate for the power lost in reflection.

### 2.7 Point Spread Function (PSF) Measurement

We used 0.1  μm blue/green/orange/dark red fluorescent beads (T7284, Invitrogen, Waltham, Massachusetts, United States) embedded in a block of 1% low melting point agarose (2.5  μl bead stock in 500 ml agarose). The spacing between prism and agarose was adjusted to correspond to the rostral part of the brain, and 250×125×50  μm stepwise stacks were acquired (1024×512  pixels lateral field of view, 250 axial steps of 0.2  μm) while the sample was illuminated by either the reflected or direct sheet for four different ExO positions. For each illumination direction and objective distance, we chose 35 beads for PSF analysis. To restrict the analysis to beads illuminated by an approximately uniform-width light sheet, all chosen beads were within 18  μm from the beam waist along the beam propagation axis (i.e., a small fraction of the 118  μm Rayleigh length). We then calculated the full width at half maximum (FWHM) from Gaussian fits to the axial and lateral intensity profiles around each bead. To measure the axial PSF of the detection optics alone, we acquired stacks by stepping the ETL optical power without changing the z position of the light sheet.

### 2.8 Assessment of Biological Images

**Fig. 2:**
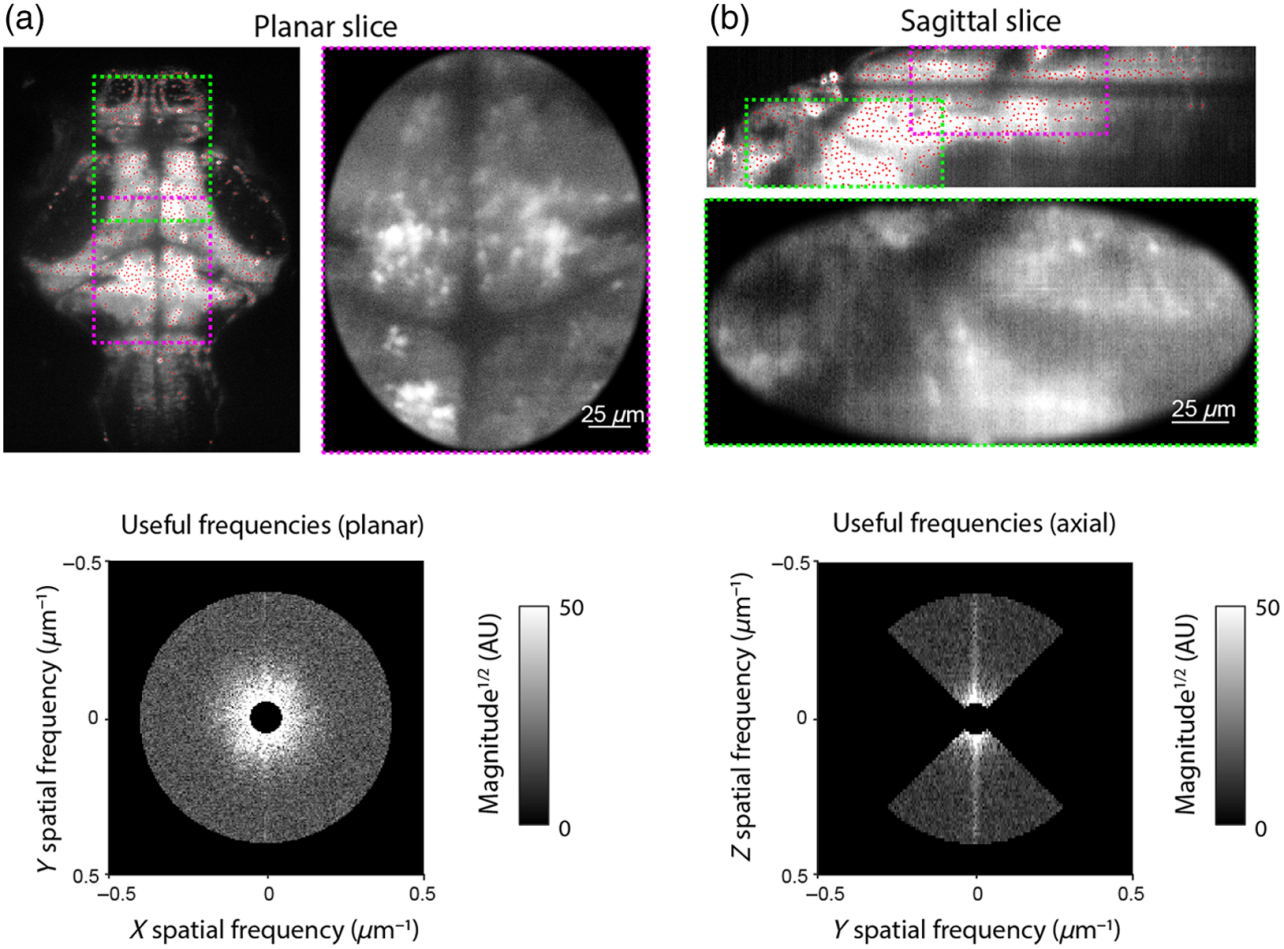
Image quality quantification. (a) Left: Raw image plane with detected ROIs indicated by red dots. Regions used for UC metric calculation are marked (direct illumination is magenta; reflected illumination is green). Right: Cropped image region following “soft” brightness truncation and windowing. Bottom: Power spectrum for the cropped image, showing biologically relevant frequencies used to calculate the planar UC. (b) Same as panel (a), for a reconstructed sagittal slice. Only spatial frequencies in the axial (Z) direction were taken for axial UC calculation.

To quantify the image quality with an emphasis on biologically relevant spatial scales, we combined two approaches: the useful contrast (UC) metric proposed by Truong et al.^13^ and automatic anatomy-based segmentation of images to detect regions of interest (ROIs) corresponding to neuronal nuclei (Fig. 2).^14^

For UC metric calculation, we first applied a “soft” truncation of pixel grayscale values to avoid bias by localized features with extreme brightness values. The pixel grey values “X” were transformed by $X^{\prime }=k\;\tanh\;\frac{X}{k}$, with the maximal brightness k chosen to be the top 0.1 brightness percentile (across the volume). Next, 200×240×100  μm sub-volumes were selected from the medial dorsocaudal or ventrorostral part of the brain [to assess direct and reflected illumination, respectively, Figs. 2(a) and 2(b)]. Individual planes were windowed with a soft-edged oval mask to avoid edge effects. To calculate planar UC, the 2D power spectrum of each plane was calculated, and the power at biologically relevant spatial frequencies (0.05 to $0.4\;{\mu m}^{-1}$, corresponding to a spatial scale of 2.5 to 20  μm) was normalized by the total power. Axial UC was obtained similarly using sagittal or coronal slices reconstructed as a cross-section along the imaging planes and taking the power at the axial direction alone [Fig. 2(b)]. To provide feedback during ExO position adjustment, planar UC was calculated online by the microscope control software during rapid automated switching between the direct and reflected illumination of a single plane.

To detect neuronal nuclei in images, every 20th image plane was automatically segmented using the algorithm proposed by Kawashima et al.^14^ This algorithm assumes that individual neurons appear as discs with radius r (we used r=2.5  μm to match the average neuron size). Briefly, it first extracts regions in the image with high local contrast, and the extracted pixels are then intensity-normalized based on a surrounding image patch (a circular region with a radius of 4r/3). The normalized image is then further smoothed by a circular kernel (with a radius of 0.5r), and ROI centroids are defined as locations of peak brightness within discs of radius r (red dots in Fig. 2). All image analysis was done in MATLAB.

### 2.9 Visual Stimulus Presentation and Response Analysis

Visual stimuli were back-projected (DLP LightCrafter E4500 MKII 385 nm UV, EKB Technologies Ltd, Bat Yam, Israel) onto a curved screen forming the wall of the imaging chamber in front of the animal, at a viewing distance of 18 mm. Visual stimuli were designed in MATLAB using the Psychophysics toolbox.^15^ Stimuli comprised 4 deg UV bright spots on a red background, moving along a circular trajectory (r=3  deg) at 5 rotations/s for 2 s, centered at an azimuth position of −40, −25, −10, 0, 10, 25, or 40 deg (pseudo-random order, five repetitions for each direction in total).

Eye position was tracked at 50 Hz under 850 nm illumination using a sub-stage GS3-U3-41C6NIR-C camera (Point Grey, Richmond, Canada). Eye movements were categorized as a convergent saccade if both eyes made nasally directed saccades within 150 ms of one another.^16^ Stimulus presentation and behavior tracking were implemented using LabVIEW and MATLAB.

For details on functional calcium imaging analysis and image registration [Fig. 6(c)], see Zylbertal and Bianco.^6^

## 3 Results

When applying mLSM, image volumes can be assembled using two types of imaging planes. For imaging the brain of larval zebrafish, dorsal planes, located above the eyes, are imaged using direct (lateral) illumination, with constant laser power during the y-mirror scan, as in conventional light-sheet imaging. However, ventral planes, which would ordinarily be occluded by the eye, are imaged with dual illumination: each exposure combines direct (lateral) as well as reflected (frontal) light-sheet excitation during a single camera frame [Fig. 1(b)].

**Fig. 3:**
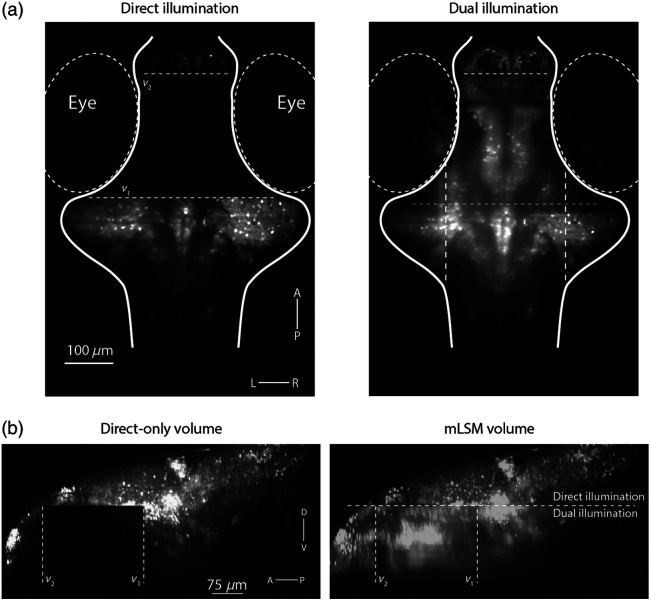
Mirror-assisted light-sheet imaging. (a) A single ventral imaging plane, acquired with conventional, direct illumination, in which the laser is off while scanning over the eyes (left) and with mLSM (dual illumination, right). (b) Sagittal maximal-intensity projection of a whole-brain volume acquired with direct-only (conventional) illumination (left) and the same volume acquired with mLSM (green).

The inspection of raw image planes acquired with dual illumination (Fig. 3, Videos 1 and 2) showed that anatomical features could be clearly resolved in the portion of the zebrafish brain that was exclusively illuminated by the reflected sheet. This region comprises 25% of the imaged brain volume ($1\times 10^{7}$ out of $3.9\times 10^{7}\;{\mu m}^{3}$) and includes 20% of all automatically segmented ROIs ($1.4\times 10^{4}$ out of $7\times 10^{4}$ ROIs, corresponding to neuronal nuclei).

### 3.1 Image Quality Considerations

For mLSM, both direct and reflected light sheets derive from the same ExO and, in our implementation, illuminate the specimen during a single camera exposure. Both features have implications for the image quality that we consider in this section.

**Fig. 4:**
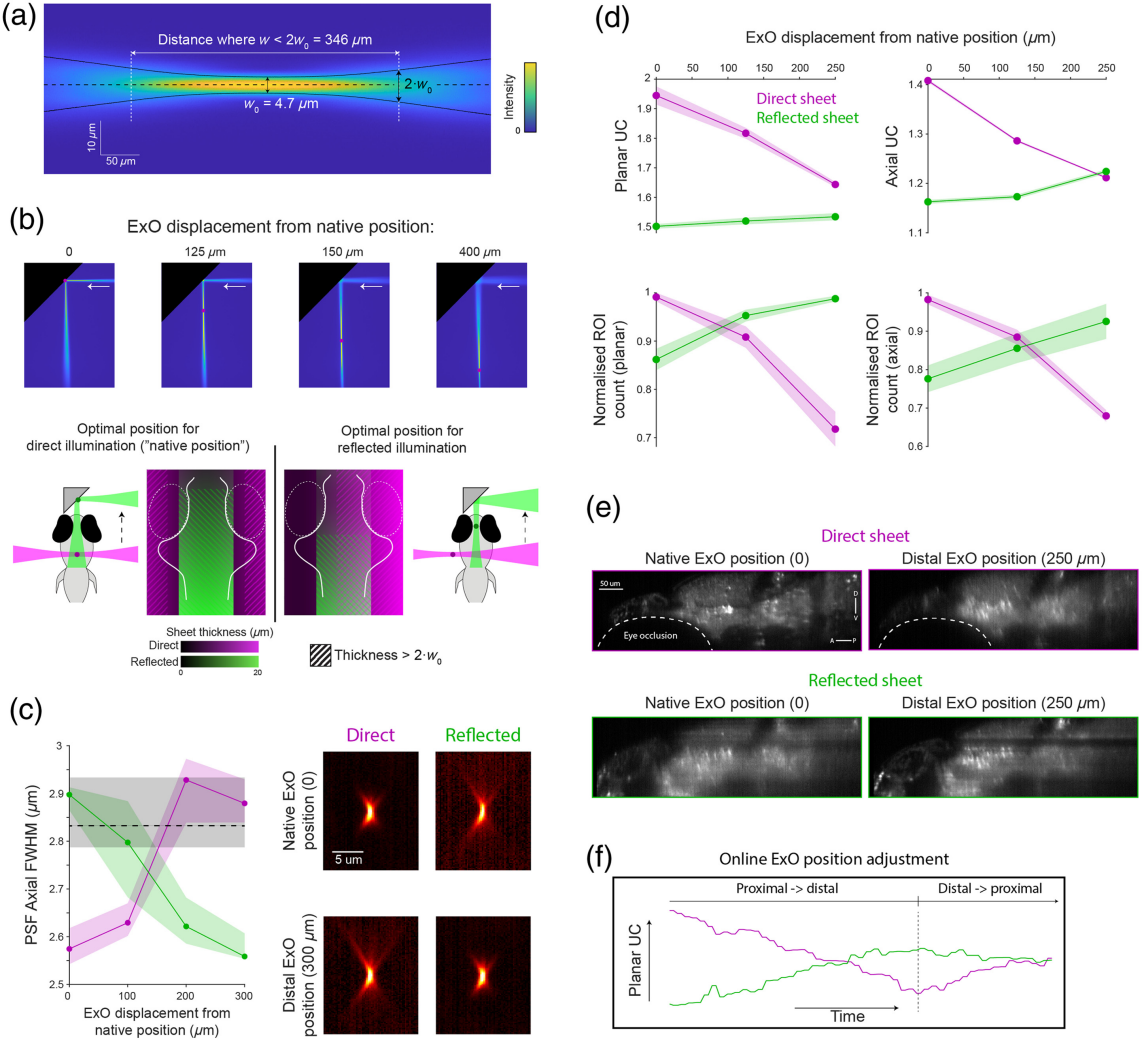
Image quality is sensitive to the geometry and position of direct and reflected beams. (a) Measured Gaussian beam properties (direct illumination of fluorescein). (b) Translating the ExO toward the specimen repositions the waist of the direct and reflected beams. Top: views of the reflected beam at four different ExO positions (red circles denote the beam waist). At the native position (“zero”), the waist of the direct beam is centered at the midline of the brain. Bottom: The trade-off between direct and reflected sheet thickness, at different ExO positions. (c) Left: PSF axial size as a function of ExO position with direct (magenta) or reflected (green) illumination (N=35 beads). The black dashed line indicates the axial PSF of the detection optics alone. Lines show a median with 40th to 60th percentiles shaded. Right: PSF X-Z profile acquired with direct (left) and reflected (right) illumination, with native (top) and distal (bottom) ExO positions (median images, gamma-corrected, γ=0.7). (d) Image quality as a function of ExO position, as measured by UC (top), or number of detected ROIs (bottom, normalized by the maximal number of ROIs detected for each illumination direction). Left: Planar quality measures, averaged across planes. Right: Axial quality measures, averaged across sagittal slices. The shaded area indicates standard error of the mean (SEM) across slices. (e) Example sagittal section acquired with direct (top) and reflected (bottom) illumination, at native (left) and distal (right) ExO positions. (f) Example of online assessment of UC while manually adjusting ExO position to achieve an approximately equal image quality for direct and reflected illumination. A, anterior; P, posterior; D, dorsal; V, ventral.

As a single ExO is used to generate both the direct and reflected light sheets, a limitation of mLSM is that the Gaussian beam profile cannot be independently adjusted for the two excitation paths. Specifically, the Gaussian beam width, w, reaches a minimal value ($w_{0}$) at the focal plane of the ExO, and the field of view (FOV) can be defined as the beam propagation distance over which the sheet is less than twice this minimal waist thickness [Fig. 4(a)]. By adjusting the excitation NA (using an iris), a compromise between $w_{0}$ and FOV must be found such that a sufficiently thin light sheet (of similar width to the biological features of interest) extends over an FOV that is adequate to cover the specimen of interest. Thus, a higher NA reduces $w_{0}$ and the FOV, whereas a lower NA increases both. In our microscope, we used a 5.0 mm iris to set the excitation NA to ∼0.06. For the resulting Gaussian beam, we measured $w_{0}=4.7\;\mu m$ (FWHM) and FOV=346  μm, similar to the theoretical values of 2.7 and 350  μm, respectively (for 488 nm excitation wavelength). With this beam geometry and the ExO at its native position (i.e., where the beam waist is centered at the midline of the brain), the direct illumination path produced a light sheet that was sufficiently thin to image zebrafish neurons across the width of the brain. However, in the reflected beam path, the additional prism-to-specimen distance (∼120  μm) means that the tissue is illuminated by a light sheet that is thicker than $2\cdot w_{0}$ [Fig. 4(b)]. A thinner reflected light sheet can be obtained by translating ExO toward the specimen, but this will shift the beam waist away from the center of the specimen in the direct illumination path.

To evaluate this trade-off, we first made point-spread function measurements to empirically verify the axial resolution obtained with direct and reflected illumination. We obtained image stacks of a field of fluorescent beads, under either direct or reflected illumination, at four different positions of the ExO. As expected, (1) the axial FWHM increased for beads illuminated by thicker parts of either beam (i.e., further away from the beam waist, $w_{0}$) and (2) translating the ExO toward the sample improved the axial resolution for the reflected light sheet but degraded it for the direct light sheet. We note that the axial PSF is bounded by the high-NA detection objective [NA = 1.0, ∼2.8  μm; dashed line in Fig. 4(c)] such that near-cellular resolution imaging is still obtained in regions of the sample far from the beam waist, using either direct or reflected illumination. Lateral PSF FWHM showed minimal variation with the ExO position (0.63 to 0.66  μm, 20 to 60th percentiles), although we note that the magnification of our system was not well suited to measure the lateral extent of beads (∼3 camera pixels/bead).

When imaging biological samples, excitation light is scattered as it propagates through tissue such that real imaging performance invariably deviates from ideal conditions. We therefore used two metrics to quantitatively assess the quality of biological images obtained with each excitation path. First, we applied the useful contrast (UC) metric proposed by Truong et al.,^13^ which measures the spatial frequency content of images at biologically relevant length scales and is a function of resolution, contrast, and signal-to-noise ratio (Sec. 2). Second, we tested how imaging conditions affect the automatic detection of putative neuronal ROIs using a contrast-based segmentation algorithm.^14^ We acquired image volumes spanning the entire larval zebrafish brain with either direct or reflected illumination at three different ExO positions; calculated the planar UC [averaged across planes, Fig. 2(a)] and the axial UC [averaged across sagittal slices, Fig. 2(b)]; and applied the segmentation algorithm on every 20th plane or sagittal slice. In agreement with theory and measured changes in PSF, both metrics showed that translating the ExO away from its “native position” improves the image quality under reflected illumination at the expense of the image quality under direct illumination [Figs. 4(d) and 4(e)]. However, in general, the image quality under reflected illumination was somewhat less sensitive to the ExO position, which might be because it is bounded by the scattering properties of the anterior tissue along the path of the reflected illumination (e.g., the animal’s jaw). This is in agreement with previous reports, in which lateral illumination of the larval zebrafish brain generated a greater proportion of cellular resolution data (92% of the imaged volume^2^) compared with anterior illumination (80%^9^).

In sum, in mLSM, a balance must be struck between the image quality obtained using direct versus reflected light sheets, which is controlled by adjusting the ExO position. For specific research applications, this balance will depend on factors such as what fraction of the sample can be accessed by each light sheet and in what regions of the sample the highest image quality is required. We also note that the relative position of the prism with respect to the sample can vary slightly from one experiment to the next. For these reasons, we implemented online UC calculation within our microscope control software (Sec. 2) to provide the experimenter with immediate feedback during manual translation of the ExO such that the image quality can be easily tuned for every experiment.

**Fig. 5:**
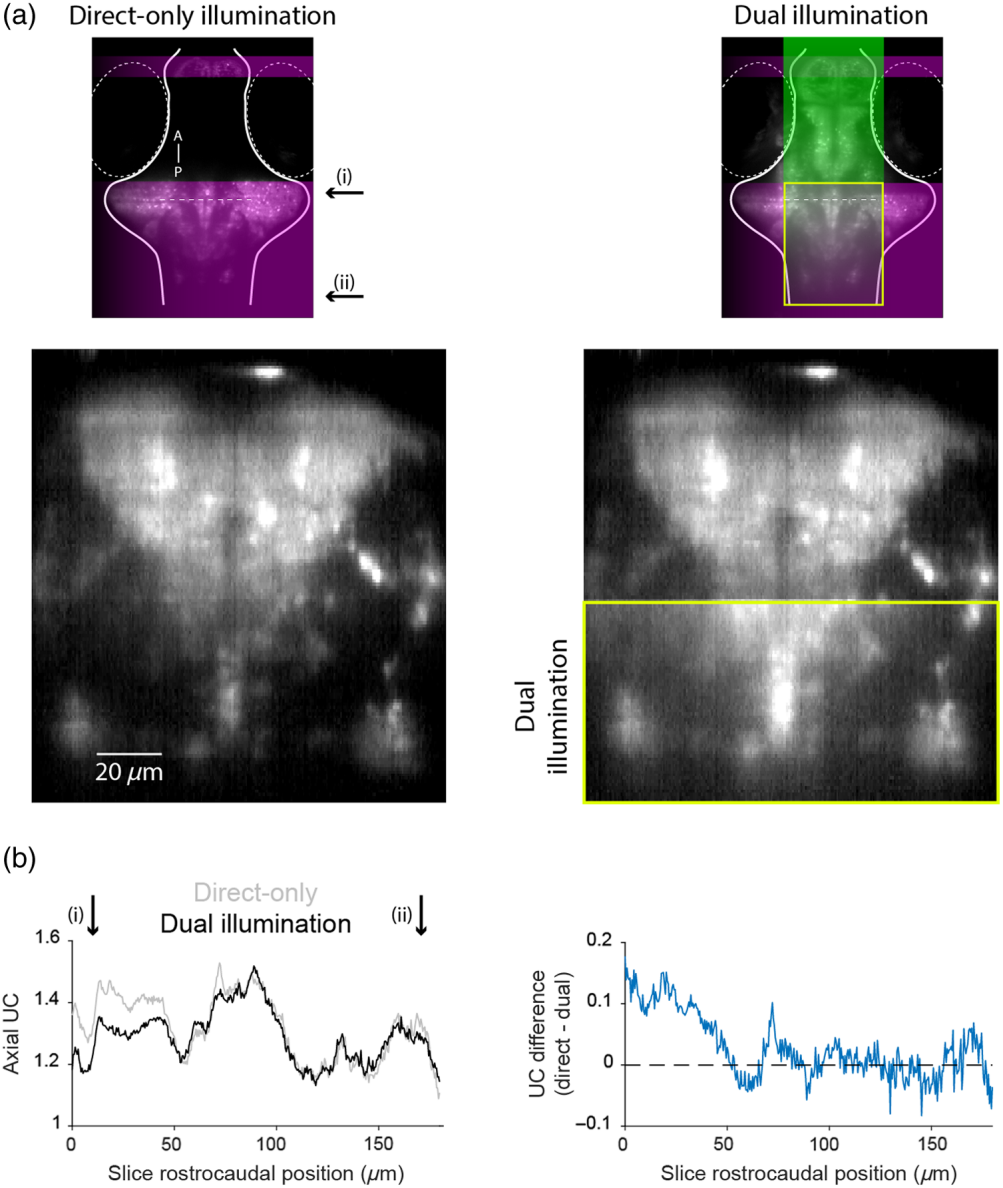
Effect of dual illumination on the image quality. (a) Top: a ventral imaging plane with direct-only (left) or dual illumination (right). Bottom: A coronal slice at the level indicated by the dashed line in the top panels. The yellow box indicates the region of overlapping illumination. (b) Left: Axial UC as a function of rostrocaudal position [from i to ii, indicated in panel (a)] within the overlap region. Right: Difference in UC between dual illumination and direct-only illumination.

In our implementation of mLSM, we chose to combine direct and reflected light-sheet illumination within individual camera frames to maximize the frame rate for functional imaging. A consequence of this is that some regions of the sample are illuminated bi-directionally, and in those regions, excitation by the thicker light sheet is expected to degrade the axial resolution. To assess this issue under routine imaging conditions, we compared UC for direct-only versus dual illumination in the region of the sample that is excited by both beam paths [yellow box in Fig. 5(a)]. Our results show that the addition of the reflected sheet had only a modest effect on axial UC, and it was restricted to around 50  μm at the rostral edge of the overlap region. We conclude that, at least for this application, the use of dual illumination has only a modest impact on the image quality while providing access to a substantial portion of the brain that is ordinarily inaccessible.

### 3.2 Visuomotor Behavior

**Fig. 6:**
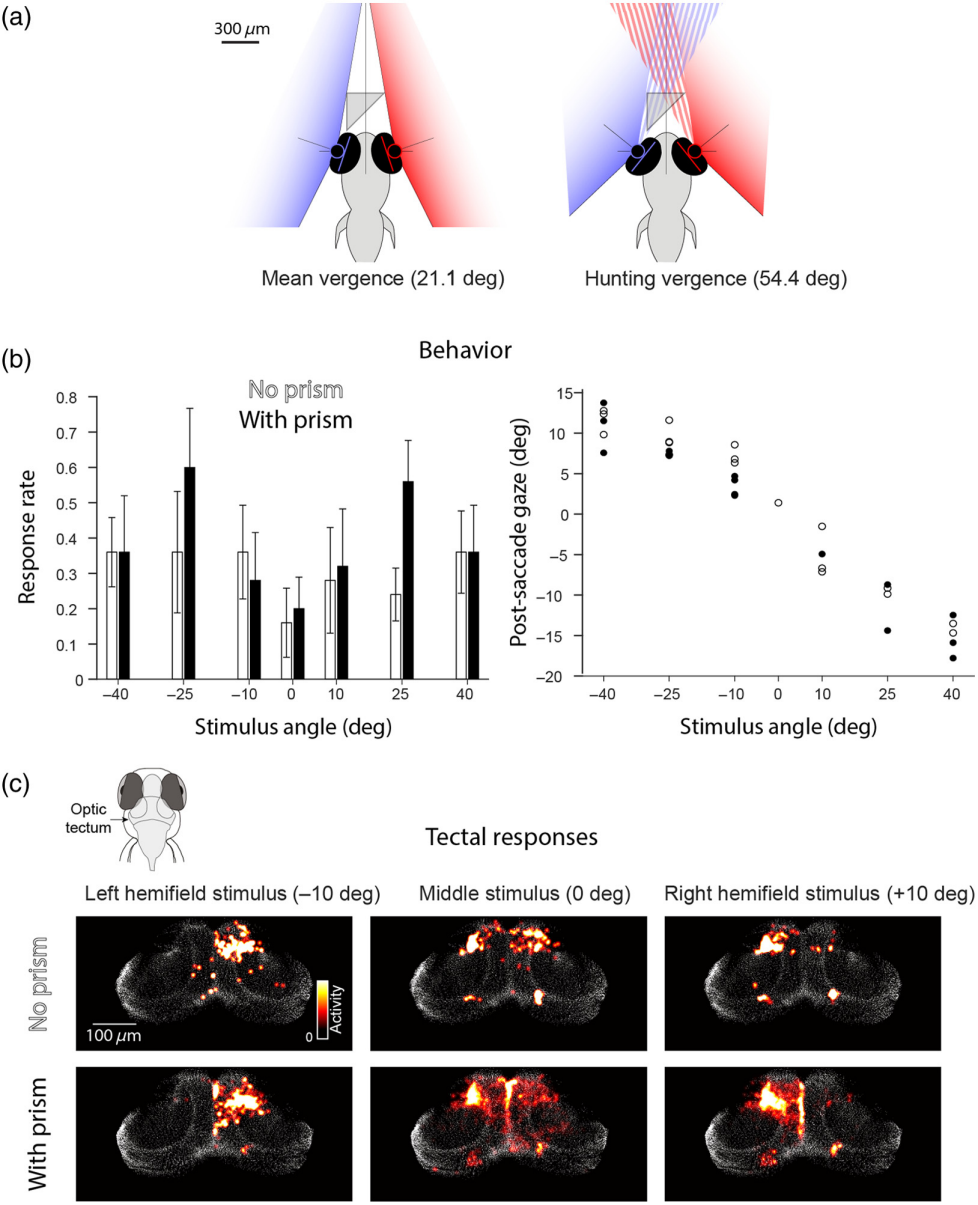
Micro-prism does not obstruct or distort the visual scene. (a) Predicted impact of the micro-prism on the visual field during normal vergence (left) and following a convergent saccade (right).^17^ (b) Left: Rate of hunting (convergent saccade) responses to rotating spot stimuli presented at different azimuth angles in the absence (white) and presence (black) of the prism (N=5 fish, error bars indicate SEM). Right: Post-saccade gaze angles in a single fish in the absence (empty circles) and presence (filled circles) of the micro-prism. (c) Neural activity in the optic tectum evoked by the presentation of −10, 0, and +10  deg stimuli for one fish in the absence (top) and presence (bottom) of the micro-prism. Each panel shows activity averaged over the 2 s of stimulus presentation (median of five repetitions).

A potential concern with mLSM is that, although the micro-prism is very small, by placing it in front of the fish, it may nonetheless obstruct the animal’s visual field, in turn interfering with the analysis of visually evoked neural activity and behavior. We calculated that, with typical prism positioning and mean ocular vergence angle (21.1 deg^17^), the prism should be just outside the visual field and therefore have only minimal effects [Fig. 6(a)]. To test this, we presented 10 larval zebrafish with prey-like moving spots centered at seven different positions across the animals’ frontal visual field (ranging from −40  deg to +40  deg azimuth) while tracking their behavior and performing calcium imaging to assess visually evoked neural activity. The protocol was repeated twice for each fish, in the presence and absence of the micro-prism.

The presence of the prism did not affect behavior or visually evoked neural activity. Specifically, the probability of visually evoked hunting responses, as identified by convergent saccades, and their directionality were comparable in the presence versus the absence of the micro-prism [Fig. 6(b)]. Furthermore, we observed similar visually evoked neural activity in the optic tectum for stimuli presented across the range of azimuth positions [Fig. 6(c)]. The prism is unlikely to influence the lower and lateral visual field, and accordingly, we did not observe a difference in the optomotor responses evoked by drifting gratings presented below the animal (data not shown). Taken together, these observations suggest that the prism does not significantly obstruct or distort the visual scene. However, we note that, when fish initiate hunting, eye convergence substantially increases the size and proximity of the binocular visual field.^16^^,^^17^ Under these conditions, we estimate that the prism occludes ∼20  deg of the visual field [Fig. 6(a)], potentially impacting experiments involving hunting sequences (Sec. 4).

## 4 Discussion and Conclusion

In this study, we demonstrated that a simple adaptation to a digitally scanned light-sheet microscope enables cellular-resolution imaging in regions of a sample that are normally occluded during unidirectional illumination. By positioning a mirrored micro-prism in front of a larval zebrafish and adapting the scanning waveforms accordingly, we were able to produce a second reflected light sheet and gain access to the 20% to 25% of brain tissue that lies between the eyes. Importantly, this simple and inexpensive upgrade can be readily implemented in existing setups, including simple open-source designs,^18^^,^^19^ by adding a single element. By avoiding the need for a second excitation objective, visual stimuli may be readily presented on a screen in front of the animal.

Although we developed this approach for functional calcium imaging in larval zebrafish, it could be used in other cases in which a second excitation path would provide access to a greater portion of the sample tissue. A key constraint to consider when adapting the method to a different sample geometry is the required width of the reflected sheet. A wider reflected sheet would require a bulkier micro-prism with a longer hypotenuse, in turn increasing the difference in the path length between the two beams and the required scan range along the y-axis. The approach that we describe is therefore best suited for cases in which a relatively thin reflected sheet is sufficient.

In our implementation, the different path lengths for the direct and reflected beams resulted in a trade-off in the image quality obtained using the two light sheets. However, when imaging real biological samples, we found that mLSM was still able to produce near-cellular resolution images, including in regions of the sample illuminated exclusively by the reflected sheet or illuminated by both. In future implementations, path length differences could be corrected by the addition of a fixed optical element that intersects part of the scanned excitation beam. For example, a block of high refractive index material attached to the prism assembly (or located in the scanning telescope) could serve to push the beam waist away from the ExO, compensating for the longer reflected beam path.^20^ Alternatively, separate frames may be acquired for direct versus reflected illumination, while the beam waist is translated using an active element, such as an ETL or a piezoelectric actuator. This latter approach could also be used to prevent degradation of the axial resolution in regions of the sample illuminated by both sheets, although we found that, in practice, this effect was minor, and we opted not to reduce the acquisition frame rates. Acquiring sequential frames also allows those using direct illumination to benefit from “light-sheet readout mode,”^21^ which is incompatible with dual illumination.

Returning to the use case of imaging neural activity during visuomotor behavior in larval zebrafish, geometrical considerations indicated that the tiny micro-prism should minimally interfere with the animals’ frontal visual field. This was confirmed by observing visually evoked behavior and tectal activity (Fig. 6). However, an occlusion of at least 20 deg from the nasal side of the visual field of each eye is likely during hunting sequences in which the eyes are converged.^17^^,^^22^ A potential workaround would be to implement mLSM using a smaller prism. A 150  μm-edged prism, for example, would still produce a sufficiently wide reflected sheet and greatly reduce occlusion of the visual field, even when the eyes are converged.

In conclusion, mLSM is a simple, versatile, and robust method for adding a second illumination axis to existing digitally scanned light-sheet microscopes. It is suited for cases in which opaque tissue prevents uniform illumination of the sample and can be used in conjunction with visual stimulus delivery and behavioral assays in larval zebrafish.

## 5 Appendix: Supplementary Video

**Video 1**: Stacks acquired with dynamic z scanning with direct-only illumination (left) and mLSM (right) (MP4, 31.3 MB [URL: https://doi.org/10.1117/1.NPh.11.3.035006.s1]).
**Video 2**: 3D maximal-intensity projection of a whole-brain volume acquired with direct-only illumination (left) and the same volume acquired with mLSM (right) (MP4, 1.25 MB [URL: https://doi.org/10.1117/1.NPh.11.3.035006.s2]).

## Supplementary Material







## Data Availability

All data in support of the findings of this paper are available within the article or as Supplementary Material.
