# Supplementary material for: Mirror-assisted light-sheet microscopy: a simple upgrade to enable bi-directional sample excitation
Source: Neurophotonics. 2024 Aug 7;11(3):035006. doi: 10.1117/1.NPh.11.3.035006 (PMC11304984; doi:10.1117/1.NPh.11.3.035006)
Supplement: Supplementary file 1 [file NPh_011_035006_SD001.pdf]

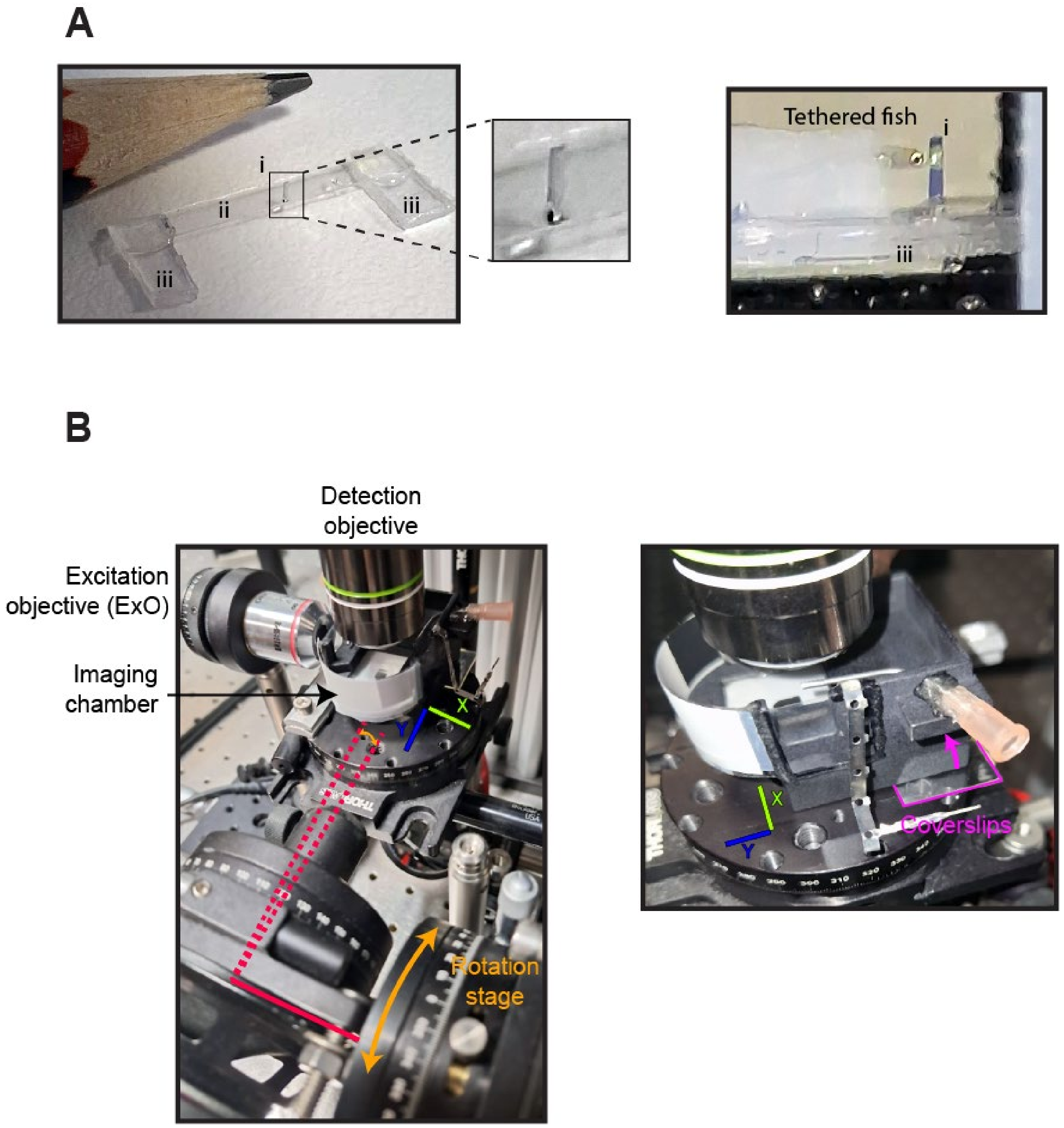


**Figure S1: Photos of mLSM components.** (**A**) Left: Micro-prism (i) attached to a glass strip (ii) and plastic bases (iii). Inset: magnified view of the micro-prism. Right: Prism assembly positioned for imaging of a tethered fish, viewed along the direction of the excitation beam. (**B**) Pitch angle adjustment methods to correct for beam slanting. Left: Rotation stage (orange double arrow) attached to the arm holding the chamber (dashed red line) for rotation about the X axis. Right: Alternatively, a stack of coverslips positioned beneath the chamber (magenta outline) can be used for tilt adjustment.
